# Site-Specific, Insertional Inactivation of *incA* in *Chlamydia trachomatis* Using a Group II Intron

**DOI:** 10.1371/journal.pone.0083989

**Published:** 2013-12-31

**Authors:** Cayla M. Johnson, Derek J. Fisher

**Affiliations:** Department of Microbiology, Southern Illinois University, Carbondale, Illinois, United States of America; Midwestern University, United States of America

## Abstract

*Chlamydia trachomatis* is an obligate, intracellular bacterial pathogen that has until more recently remained recalcitrant to genetic manipulation. However, the field still remains hindered by the absence of tools to create selectable, targeted chromosomal mutations. Previous work with mobile group II introns demonstrated that they can be retargeted by altering DNA sequences within the intron’s substrate recognition region to create site-specific gene insertions. This platform (marketed as TargeTron™, Sigma) has been successfully employed in a variety of bacteria. We subsequently modified TargeTron™ for use in *C. trachomatis* and as proof of principle used our system to insertionally inactivate *incA*, a chromosomal gene encoding a protein required for homotypic fusion of chlamydial inclusions. *C. trachomatis incA*::GII(*bla*) mutants were selected with ampicillin and plaque purified clones were then isolated for genotypic and phenotypic analysis. PCR, Southern blotting, and DNA sequencing verified proper GII(*bla*) insertion, while continuous passaging in the absence of selection demonstrated that the insertion was stable. As seen with naturally occurring IncA^−^ mutants, light and immunofluorescence microscopy confirmed the presence of non-fusogenic inclusions in cells infected with the *incA*::GII(*bla*) mutants at a multiplicity of infection greater than one. Lack of IncA production by mutant clones was further confirmed by Western blotting. Ultimately, the ease of retargeting the intron, ability to select for mutants, and intron stability in the absence of selection makes this method a powerful addition to the growing chlamydial molecular toolbox.

## Introduction

The *Chlamydia* are obligate, intracellular bacterial pathogens that infect humans as well as a wide variety of animals including economically important poultry and livestock [1]. Of paramount importance to human health is *C. trachomatis*, the causative agent of sexually transmitted infections and trachoma, the former of which remains the leading reportable bacterial infection both in the United States and world-wide [2], [3]. These pathogens share a unique physiology in which the bacteria undergo a biphasic developmental cycle initiated when the extracellular, non-replicative form known as the elementary body (EB) binds to the surface of a susceptible eukaryotic cell [4]. The EB is then internalized where it resides in a host derived membrane vacuole called an inclusion. Within the inclusion, the EB differentiates into the replicative form known as the reticulate body (RB). During growth within the inclusion, the bacteria secrete a variety of effecter proteins using a type III secretion system [5]. Some of these proteins, designated as inclusion membrane proteins (Incs), are inserted into the inclusion membrane where they function in nutrient acquisition, inclusion growth and fusion, and protection from host intracellular immune attack [6]. After approximately 15 hours, depending upon the species, the RBs begin to convert back to EBs and are released from the cell either by cell lysis or inclusion extrusion after 40–72 hours [7].

Research focusing on these highly significant pathogens has been largely hindered by the paucity of genetic methods with which to manipulate these organisms. Stable introduction and maintenance of foreign DNA in *Chlamydia* spp. was first reported by Binet and Maurelli using *C. psittaci* as the recipient of an altered 16S rRNA allele that was incorporated into the bacterial chromosome [8]. Genetic manipulation of *Chlamydia* was further advanced by the development of a chemical transformation protocol by Wang *et al*. demonstrating the ability of *C. trachomatis* serovar L2 to be stably transformed with a modified version of the serovar E cryptic plasmid [9]. This study provided evidence that foreign genes could be maintained and functionally expressed in *C. trachomatis* providing the foundation for numerous groups to begin assessing plasmid gene function in chlamydial growth and infection and to develop cryptic plasmid based expression vector platforms [10], [11], [12], [13]. Finally, work by Alan Hudson and colleagues has extended the utility of plasmid-based genetic tools to *C. pneumoniae* [14].

While current methods allow for the creation of plasmid gene knockouts (performed *in vitro* and then reintroduced into *Chlamydia*) and plasmid-based gene knock-in approaches, the field still lacks tools to create targeted, selectable chromosomal mutations. Recently, chemical mutagenesis has been employed for use in forward genetic approaches and TILLING screens to obtain chromosomal mutants [15], [16]. However, chemical mutagenesis approaches often result in undesired second site mutations, and the mutations in genes of interest are still random in nature. These short comings often require the creation of multiple mutants, performance of whole genome sequencing, and/or back crossing of mutants with a wild-type parental strain to obtain clean mutant backgrounds to allow a phenotype to be mapped to a particular genotype. Although effective, these approaches are costly and time-consuming. With these limitations in mind, we set out to develop an alternative method to create selectable, targeted chromosomal mutations.

Mobile group II introns exist in approximately 25% of bacterial genomes (although none have been identified in *Chlamydia*) and move between genes via a retrotransposition mechanism typically requiring help from an intron encoded protein (IEP) possessing RNA maturase, endonuclease, and reverse transcriptase activity [17]. Intron target recognition is based upon sequence recognition between the intron (designated as EBS2, EBS1, and δ) and the target gene (designated as IBS2, IBS1, and δ’), resulting in insertion of the intron into the target gene between the IBS1 and δ’ sites. During the normal intron “life-cycle”, the intron remains stably inserted into the DNA, but is spliced out of the RNA after transcription, resulting in formation of the wild-type transcript and no loss of gene function. Extensive work with the intron Ll.LtrB from *Lactococcus lactis* demonstrated that the intron can be targeted to genes of interest by changing the EBS1, EBS2, and δ intron sequences, allowing base pair recognition and insertion into new target genes [18]. Optimal base pairing is predicted by a proprietary algorithm and intron retargeting is carried out by PCR-based modification of the intron. To prevent restoration of gene function via RNA splicing post-transcription, the Ll.LtrB IEP *ltrA* gene is removed from the intron and expressed in trans. Removal of the IEP allows the intron to carry alternative genes such as selection cassettes. Both intron and *ltrA* are supplied to the bacterium on a plasmid and expression of the intron and *ltrA* leads to insertion of the intron into the targeted gene. After intron insertion, the intron donor plasmid is cured, leading to the absence of the LtrA and prevention of intron splicing, resulting in an insertional gene inactivation. This technology is marketed by Sigma as TargeTron™.

As the TargeTron™ system has been successfully employed in a variety of Gram positive and negative bacteria (including the obligate, intracellular bacterium *Ehrlichia chaffeensis*), we hypothesized that this technology should allow for targeted, selectable gene inactivation in *Chlamydia* [19], [20], [21], [22], [23], [24]. To test this hypothesis, we modified the base TargeTron™ vector platform by placing a chlamydial promoter upstream of the intron and inserting the *bla* gene into the intron to allow for intron expression and ampicillin selection in *C. trachomatis* serovar L2. As proof of principle, we used our system to inactivate *incA*. IncA is an inclusion membrane protein hypothesized to be involved in homotypic inclusion fusion when a cell is infected with more than one EB (each EB initially enters and resides within its own inclusion) [25], [26]. Consistent with previous studies of naturally occurring IncA^−^ mutants, the *incA*::GII(*bla*) mutants obtained in our study exhibited a non-fusogenic inclusion phenotype when cells were infected at a multiplicity of infection (MOI) greater than one. Genotype analysis confirmed a single intron insertion in the predicted location and Western blot analysis demonstrated the loss of IncA production by these mutants. Finally, maintenance of the intron insertion in mutants following multiple passages in the absence of selection indicated that the intron insertion is stable in *C. trachomatis*. Collectively, the ease of retargeting the intron, ability to select for mutants, and stability of intron insertion makes this an ideal method for generating chromosomal mutations in *C. trachomatis* and, to the best of our knowledge, the *incA*::GII(*bla*) mutant is the first report of a site-specific chromosomal gene inactivation in *Chlamydia*.

## Results

### Modification of the TargeTron™ System for Use in *C. trachomatis*

The base TargeTron™ vector pACD4K-C was chosen for modification for use in *C. trachomatis* (Figure S1A). This plasmid contains an *Escherichia coli* origin of replication, a *cat* marker for chloramphenicol selection, and the requisite intron genes including the GII intron, a retrotransposition-activated kanamycin resistance cassette (RAM), and the *ltrA* gene required for production of the LtrA protein which is essential for intron insertion. We first removed the RAM cassette and replaced it with the *bla* gene from pGFP::SW2 [9]. To ensure expression of the GII intron and *ltrA*, the predicted promoter region of CTL0655 (the 264 bp region upstream of the ATG codon) was cloned between the vector-encoded T7 promoter and the 5′ exon site of the intron. Finally, the intron was retargeted for *incA* using primer sequences obtained from the TargeTron™ algorithm that: 1) resulted in the lowest E-value (E-values less than 0.5 should result in efficient intron targeting) and 2) resulted in an insertion site closest to the 5′ ATG of the *incA* ORF (Figure S2) creating pDFTT3 (Figure S1B). Prior to attempting *incA* inactivation in *C. trachomatis*, the *incA* gene was cloned into the *E. coli* GST expression vector pGEX-6p-1 to test intron targeting (using a pDFTT3Δ*bla* construct) in a host where TargeTron™ has been previously shown to function [18]. Colonies carrying a disrupted *incA* gene were obtained and DNA sequencing verified that the intron had inserted in the algorithm-predicted location (data not shown).

### Selection of *incA*::GII(*bla*) Mutants

**Figure 1:**
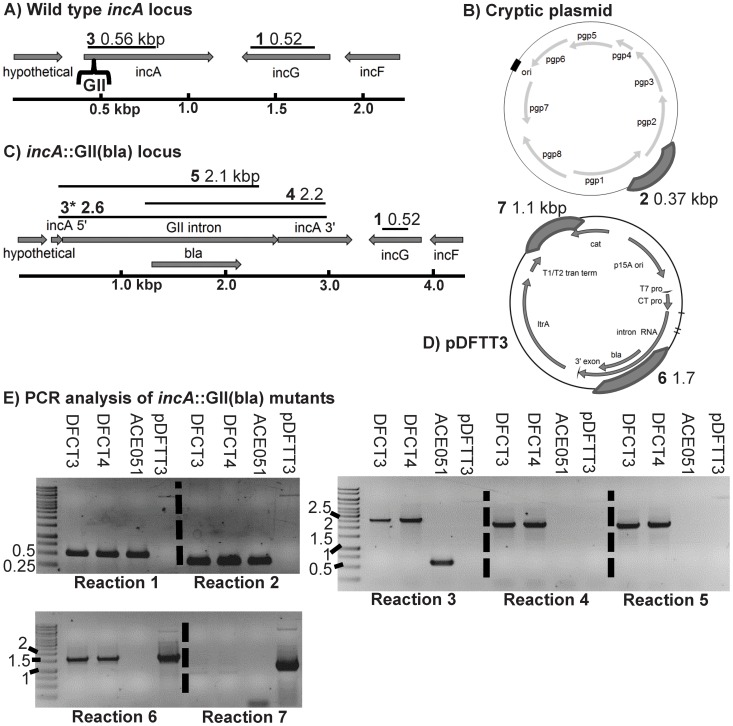
PCR verification of *incA*::GII(*bla*) clones plaque purified with or without ampicillin selection. PCR was performed using genomic DNA from the wild type ACE051 strain and the plaque purified strains DFCT3 and DFCT4 to assess intron insertion, loss of the intron donor plasmid, and maintenance of the chlamydial cryptic plasmid. Primers were designed to amplify regions of the *incA* locus, cryptic plasmid, intron, and pDFTT3 (the intron donor plasmid). Wild type and mutant *incA* locus maps and plasmid maps are shown in panels A–D. Expected PCR product sizes (in kbp) are shown on each map with the PCR reaction and region amplified represented by black bars. The predicted GII(*bla*) intron insertion site is shown in panel A with brackets. PCR reactions and primers are detailed in Tables S2 and S3. PCR results are shown in panel E. Products were separated on 1% agarose gels and stained with ethidium bromide. Gels were visualized using UV transillumination and photographed. Images have been reversed to aid in product visualization. The template used in the PCR reaction is listed at the top of each lane. Reactions performed are shown at the bottom of each gel. Molecular weight markers were loaded in lane 1 of each gel and the marker sizes are listed in kbp to the left of each gel.

ACE051 (a plaque-purified *C. trachomatis* serovar L2 strain) EBs were transformed with pDFTT3 using the calcium chloride transformation method and mouse fibroblast L2 cells were then infected via centrifugation (considered Passage 0, P_0_). Ampicillin selection was applied 12 hours post infection (PI) and EBs were harvested at 40–44 hours PI. Transformants were expanded under selection for four additional passages prior to clone isolation via plaque purification. Normal inclusions (inclusions containing non-aberrant bodies) were readily observed at P_2._ Upon P_4_ expansion, MOIs exceeded one and non-fusogenic inclusions characteristic of naturally occurring IncA^−^ mutants were observed under light microscopy [25]. P_4_ transformants were titered using the IFU assay and genotyped to assess *incA* disruption using PCR reactions 3 and 6, amplifying *incA* or GII(*bla*), respectively (see Figure 1 for loci maps). P_4_ was then used to obtain plaque-purified clonal isolates for further genotype and phenotype analyses.

For clone isolation, P_4_ transformants were plaqued either with or without ampicillin selection for 14 days. Plaques were picked and expanded in L2 cells grown in 6 well culture dishes. EBs were then harvested and stocked in SPG and one half of the stocks were then processed for PCR genotyping to confirm the presence of *C. trachomatis*, the *incA*::GII(*bla*) genotype, and intron orientation relative to *incA*. All plaques were found by PCR to contain the expected gene disruption regardless of the presence or absence of ampicillin selection during plaquing (six non-ampicillin-selected and eight ampicillin-selected plaques were assayed) (Figure S3). One ampicillin and one non-ampicillin selected isolate, DFCT3 and DFCT4, were chosen for further expansion and characterization. Expansion of DFCT3 and DFCT4 for genotype and phenotype characterization was performed with or without selection, matching the respective plaquing conditions. Despite being plaqued and expanded in the absence of ampicillin selection, DFCT4 maintained resistance to ampicillin equal to that of DFCT3 continually passaged with ampicillin (Figure S4), consistent with PCR results indicating the presence of the GII(*bla*) cassette in DFCT4 after expansion (Figure 1).

Overall, mutagenesis was successful on three of six attempts with each successful trial expanded to P_4_ for PCR genotyping, and phenotyping via microscopy. All P_4_ expansions showed the expected PCR results and non-fusogenic inclusion phenotype (data not shown). Mutagenesis attempts were performed using independently prepared buffers, EB stocks, and plasmid preparations. DFCT3 and DFCT4 were from the first successful trial.

### Genotypic Analysis of DFCT3 and DFCT4 (*incA*::GII(*bla*) Clones)

PCR using *incA*-specific primers flanking the predicted GII(*bla*) insertion site confirmed disruption of *incA* in DFCT3 and DFCT4 (Figure 1, Reaction 3), visualized by the shift in the product size of the wild type strain ACE051 versus the DFCT3 and DFCT4 mutants. Primers linking *incA* to GII(*bla*) demonstrated the sense orientation of the intron relative to *incA* (Figure 1, Reactions 4 and 5), as predicted by the TargeTron™ algorithm. PCR also was performed to ensure that: 1) the chlamydial cryptic plasmid was not lost during passage or introduction of a foreign plasmid (Figure 1, Reaction 2) and 2) that the intron donor plasmid pDFTT3 had been lost during passage (Figure 1, Reaction 7). Finally, PCR performed using *incG*-specific primers (located 3′ to *incA*) indicated that the region next to *incA* had not been affected during insertion (Figure 1, Reaction 1).

To more finely map the insertion, the *incA* locus was amplified from ACE051, DFCT3, and DFCT4 and cloned into pUC18 for analysis via Sanger sequencing (sequencing results are shown in Figure S5). Identical GII(*bla*) insertions were found in DFCT3 and DFCT4 (inserting at *incA* ORF position 108), matching the predicted insertion site. This insertion results in a premature stop codon at amino acid position 48, leading to a truncated protein of 47 amino acids compared to the wild type protein of 273 amino acids (Figure S2). Additionally, due to the frameshift mutation resulting from the intron insertion, only the first 36 amino acids match the wild type IncA sequence.

**Figure 2:**
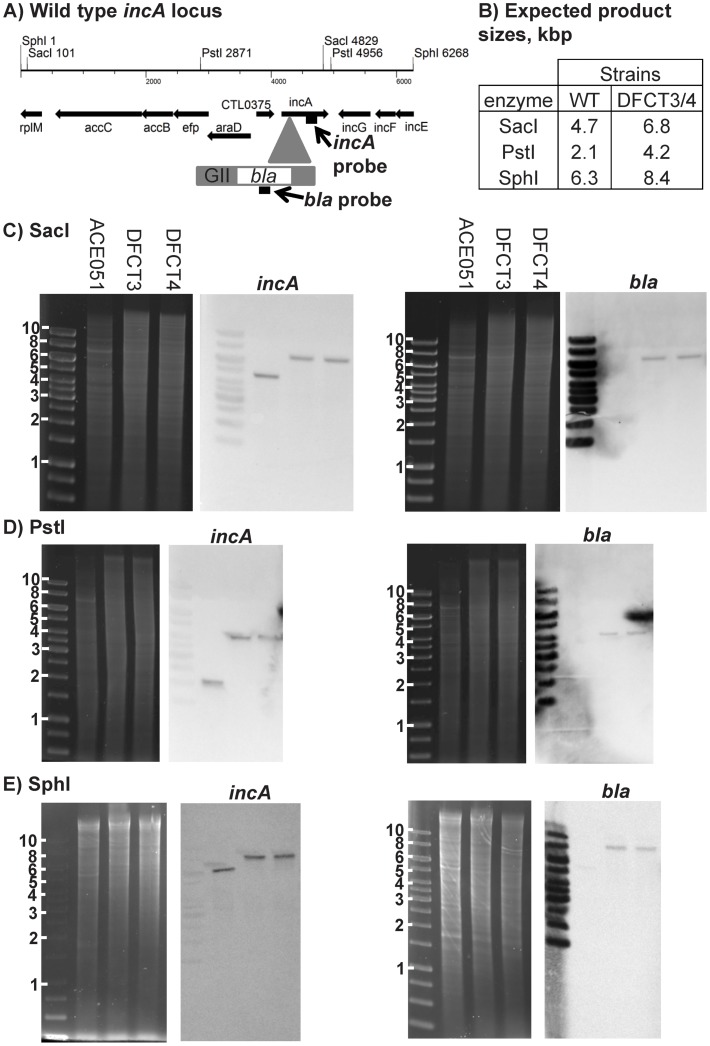
Southern blot characterization of intron insertion frequency in *incA*::GII(*bla*) clones. Southern blotting was performed using DIG-labeled probes targeting the 3′ region of *incA* or the *bla* gene located within the intron. Probe locations, restriction site locations, and expected fragment sizes are shown in panels A and B, respectively. Genomic DNA was digested with either SacI, PstI, or SphI, separated on agarose gels, stained with ethidium bromide, and viewed under UV transillumination. DNA was then transferred to nylon membranes and probed with DIG-labeled *incA* or *bla* probes. Probes were detected using anti-DIG-antibodies conjugated to alkaline phosphatase and visualized with colorimetric substrate. DNA gels and their respective Southern blots are shown for each restriction enzyme used. Molecular weight markers and sizes (in kbp) are shown to the left of each DNA gel. DNA sources are shown at the top of each DNA gel and the probe used for the respective blot is shown above each blot.

We next performed Southern blots using *incA* specific and *bla* specific probes to further assess intron insertion effects on the *incA* locus and to ensure that only a single insertion event had occurred. Genomic DNA from ACE051, DFCT3, and DFCT4 was digested with SacI, PstI, or SphI and separated on 0.7% agarose gels. The DNA was then transferred to nylon membranes and probed with DIG-labeled probes. Ethidium bromide stained gels and the corresponding Southern blots are shown in Figure 2. Predicted fragment sizes were based upon the *C. trachomatis* 434/Bu genome sequence. The *incA* probe hybridized with fragments of the expected sizes and showed a 2 kb upward shift for DFCT3 and DFCT4, consistent with the presence of GII(*bla*) in these strains. Only DFCT3 and DFCT4 were positive when probed with the DIG-*bla* probe and the fragments matched the size of the *incA*-probed samples, as predicted by the restriction map. The *bla* probe hybridized with a single DNA fragment for DFCT3 and DFCT4 when digested with each of the three restriction enzymes, confirming the presence of a single intron insertion in the mutants.

### DFCT3 and DFT4 are IncA^−^ Negative

Genotypic analysis of plaque isolates obtained in the absence of selection (particularly DFCT4, which was plaqued and expanded in the absence of selection) indicated that the *incA*::GII(*bla*) insertion was stable. Insertion stability was further supported by ampicillin minimal inhibitory concentration analysis of DFCT3 and DFCT4 in a plaque assay format (Figure S4). As such, phenotype assays with DFCT3 and DFCT4 were routinely performed in the absence of ampicillin selection to allow for growth conditions mirroring those used for the wild-type, ampicillin-sensitive parental strain ACE051.

**Figure 3:**
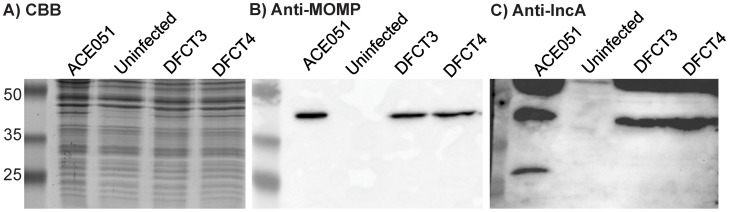
IncA Western blot analysis of the *incA*::GII(*bla*) mutants. Cells were grown until confluent and then infected at an MOI of ∼10 with ACE051, DFCT3, or DFCT4 to assess the production of IncA. After 24 hours, infected (and mock infected) cells were lysed with Laemmli buffer and loaded onto 12% SDS-PAGE gels. Gels were then either stained with Coomassie Brilliant Blue (A) or transferred to nitrocellulose for anti-MOMP blotting (B) or anti-IncA blotting (C). Expected protein sizes are 42.5 kDa (MOMP) and 30.3 kDa (IncA). Molecular weight markers are shown in lane one in each panel and marker sizes in kDa are listed to the left of panel A. Blots are representative of three independent experiments.

To confirm the loss of IncA production in DFCT3 and DFCT4 due to premature truncation of *incA* (Figure S2), Western blot analysis was carried out using anti-IncA antibodies. L2 cells were infected at an MOI of ∼ten or mock infected and grown for 24 hours. Cells were assessed for inclusion phenotype prior to sample processing. Both DFCT3 and DFCT4 showed non-fusogenic inclusion clusters whereas ACE051-infected cells typically exhibited single inclusions that were much larger in size than the DFCT3 and DFCT4 inclusions (data not shown). Cells were then lysed in Laemmli buffer, lysates were run on SDS-PAGE gels, and subsequently processed for staining with Coomassie Brilliant Blue (CBB), anti-MOMP Western blotting, or anti-IncA Western blotting. CBB staining confirmed equal sample loading (Figure 3A), while anti-MOMP (the chlamydial major outer membrane protein) Western blotting with a mouse monoclonal antibody confirmed the presence of *C. trachomatis* in the infected samples by the presence of a single band of ∼40 kDa (Figure 3B, predicted MOMP mass is 42.5 kDa). IncA Western blotting with a rabbit polyclonal anti-IncA antibody revealed the presence of a band of ∼30 kDa in the ACE051-infected cells that was absent in the DFCT3 and DFCT4 infected cells (Figure 3C, the predicted mass of IncA is 30.3 kDa).

### Altered Inclusion Phenotype of DFCT3 and DFCT4

**Figure 4:**
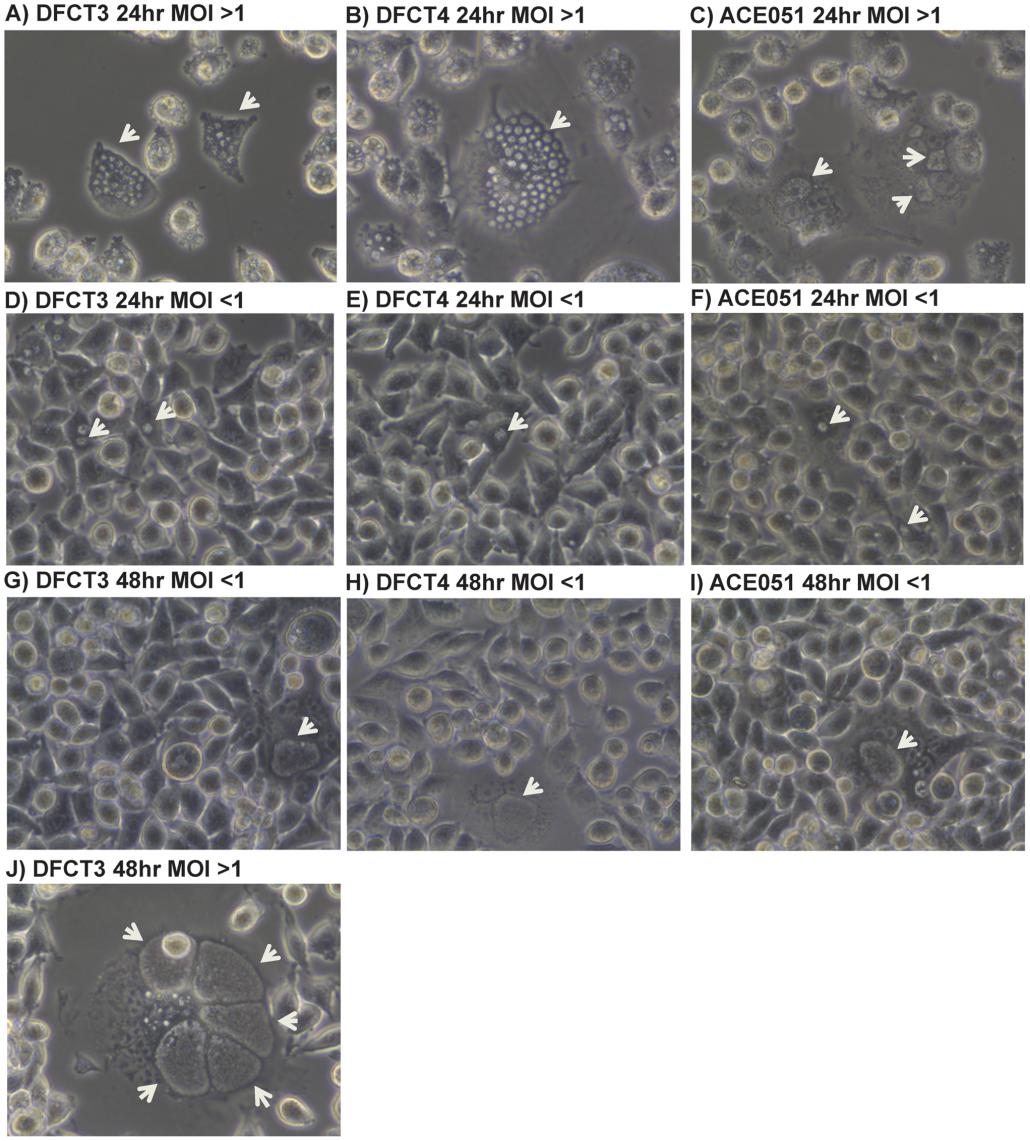
Formation of non-fusogenic inclusions by *incA*::GII(*bla*) mutants in a cell culture infection model. Cells were infected at an MOI of ∼10 (panels A–C), ∼5 (panel J), or ∼0.1 (panels D–I) with ACE051, DFCT3, or DFCT4 and viewed under 400× phase contrast at 24 (panels A–F) or 48 hours (panels G–J) post infection. White arrows designate the location of inclusions. Images are representative of three independent experiments.

Alterations in inclusion phenotype became apparent upon P_4_ expansion, in which infected cells presented with multiple inclusion-like structures. This phenotype was consistent with previous studies using naturally occurring IncA^−^ mutants obtained from clinical samples [25], [27]. To confirm this phenotype, cells were infected with DFCT3 or DFCT4 at MOIs greater than one (between five and ten) or less than one to compare the wild type and mutant inclusion phenotypes under phase contrast light microscopy. When cells were infected at an MOI of greater than one with DFCT3 and DFCT4, multiple inclusions were observed at both 24 and 48 hours PI (Figure 4A, B, and J). In contrast, cells infected with ACE051 at an MOI of greater than one primarily possessed a single inclusion (Figure 4C). Under conditions where the MOI was greater than one, the ACE051 inclusions also were larger than the inclusions formed by DFCT3 and DFCT4 at similar MOIs. All inclusions contained easily observable “moving” particles characteristic of healthy inclusions. When cells were infected with ACE051, DFCT3, or DFCT4 at an MOI of less than one, only single inclusions were present in infected cells (Figure 4D–I). The inclusion sizes were similar for DFCT3, DFCT4, and ACE051 at both 24 hours and 48 hours post infection under these infection conditions.

**Figure 5:**
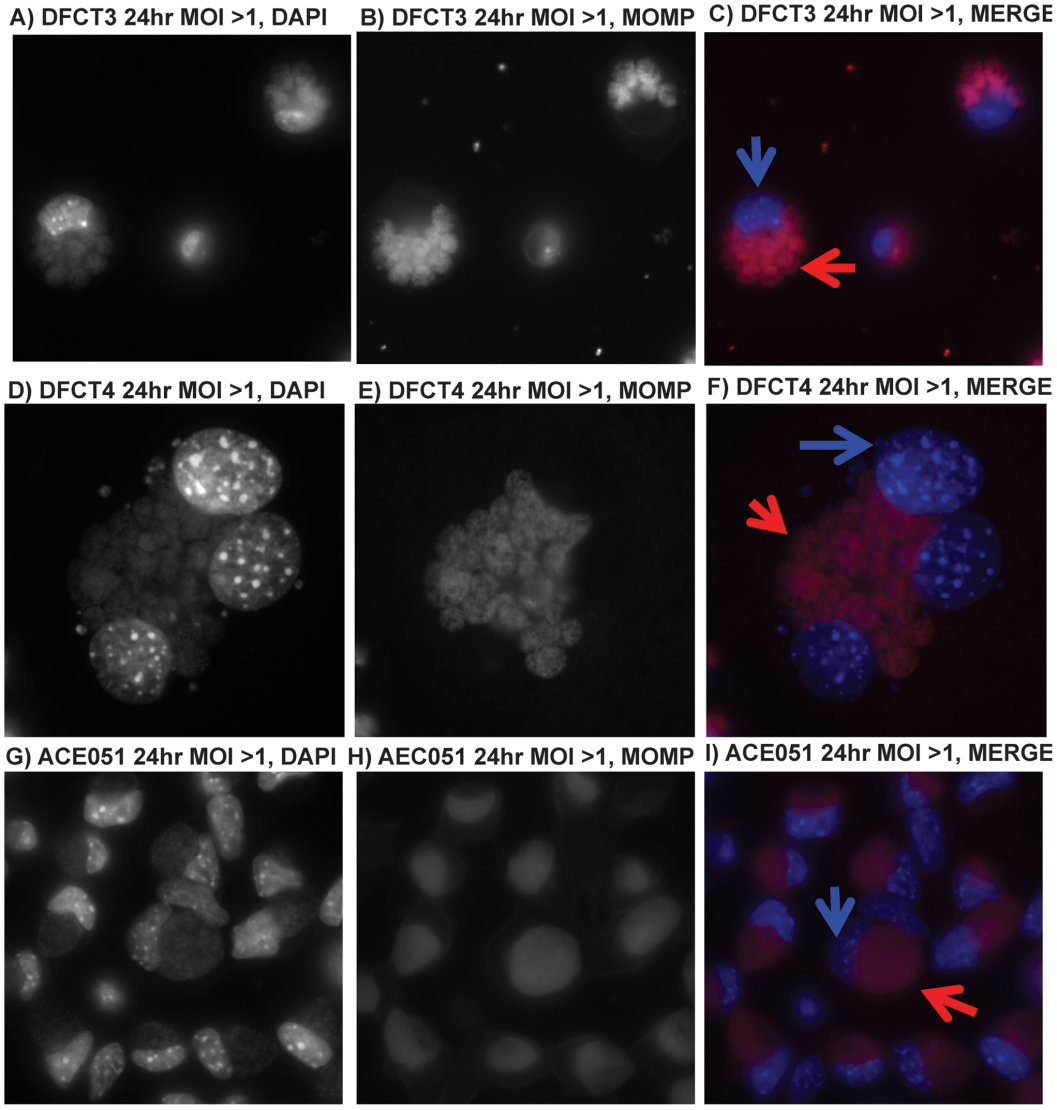
Immunofluorescence analysis of *incA*::GII(*bla*) mutants. Cells were infected at an MOI of ∼10 with ACE051, DFCT3, or DFCT4. After 24 hours post infection, cells were fixed for immuno-probing with mouse anti-MOMP antibodies to detect *Chlamydia*, panels B/E/H (visualized with a goat anti-mouse Texas Red conjugated secondary antibody), and stained with DAPI, panels A/D/G. For image overlay analysis, anti-MOMP images were false colored red and DAPI images were false colored blue. The resulting overlays are shown in panels C/F/I. Blue arrows in panels C/F/I highlight cell nuclei while red arrows highlight inclusions. All images were taken under oil immersion at 630× using a fluorescent microscope. Infections and immuno-processing were performed three times.

Cells infected at MOIs greater than one also were processed for immunofluorescence microscopy. Cells were fixed and permeablized at 24 hours post infection, probed with a mouse monoclonal anti-MOMP antibody (followed by incubation with a Texas Red conjugated anti-mouse antibody) to detect *C. trachomatis* and then stained with DAPI to detect cell nuclei and *C. trachomatis*. Mirroring results from phase contrast microscopy experiments, both MOMP immunodetection and DAPI staining of DFCT3/DFCT4-infected cells revealed the presence of multiple, non-fused inclusions (Figure 5A–F). In contrast, ACE051-infected cells primarily contained single inclusions that were larger in size than the individual inclusions in the mutant-infected cells (Figure 5G–I).

## Discussion

The chlamydial research community has made great strides in developing methods to genetically modify these important bacterial pathogens. While still technically challenging, foreign DNA can now be routinely introduced and stably maintained within *C. trachomatis* and *C. pneumoniae*. Use of cryptic plasmid-based vector platforms for functional expression of foreign genes including the *bla* and *cat* antibiotic selection cassettes, multiple fluorescent proteins, and the *tet* ON/OFF promoter system has already and will continue to facilitate research not previously feasible in *Chlamydia* [9], [10], [11], [12], [13], [28]. In addition, gene silencing with dendrimer-delivered anti-sense RNA has been used to modulate gene expression in *C. trachomatis* and chemical mutagenesis strategies have been developed for use in forward genetic and TILLING approaches [14], [15], [16], [29]. The last major hurdle remaining for chlamydial genetics was the ability to manipulate the chromosome through allelic exchange mutagenesis and/or selectable, insertional gene inactivation methods such as transposon mutagenesis or group II intron-based approaches. Our TargeTron™-based platform serves as the first step in providing a tool to manipulate the chlamydial chromosome in a selectable and targeted manner.

As proof of principle, we targeted the *incA* gene in our study due to the existence of naturally occurring IncA^−^ mutants isolated from clinical samples, indicating that a *C. trachomatis incA*::GII(*bla*) mutant would be viable [25], [27], [30]. Furthermore, while the *incA* locus is present near other *inc* genes (*incDEFG*), its orientation is opposite to these genes and transcription analysis demonstrates that it is regulated independently [31]. This data suggests that polarity effects from a disrupted *incA* gene should be absent or minimal, allowing the mutant phenotype to be attributed to the *incA*::GII(*bla*) mutation. While previous studies using naturally occurring IncA^−^ mutants, recombinant IncA protein/liposome fusion approaches, and anti-IncA antibody microinjection experiments have made a strong case for the role of IncA in inclusion fusion, the absence of isogenic mutant and parental strains has made it difficult to conclusively link *incA* inactivation with the lack of inclusion fusion [25], [27], [32], [33]. Data from our study demonstrating non-fusogenic inclusions in a cell infected at an MOI of greater than one with the IncA^−^ mutants compared to the isogenic parental strain (Figures 4 and 5) confirms that IncA is necessary for inclusion fusion. Attempts to complement DFCT3 with *incA* in trans and to assess the effect of *incA* inactivation on chlamydial growth and infectivity are currently ongoing.

In our hands, the TargeTron™ system successfully generated *incA*::GII(*bla*) mutants in 50% of the trials. Trials were performed using different EB preparations, as well as independently prepared buffers and plasmid DNA preparations, indicating that the method is fairly robust. In failed trials, it is unclear whether lack of mutants was due to failure to transform EBs or absence of intron production and/or insertion. However, as our lab has had similar success rates when transforming pGFP::SW2 into *C. trachomatis* serovar L2, we infer that failed trials were most likely due to lack of transformation. For all successful trials, genotype and phenotype results were identical to the results from the fully-characterized mutants DFCT3 and DFCT4, indicating that the mutagenesis itself is reproducible. Sequencing of the *incA*::GII(*bla*) locus from DFCT3 and DFCT4 confirmed the expected intron insertion site in *incA*, demonstrating that the TargeTron™ algorithm is functional for chlamydial genome sequences and that intron/LtrA targeting is not altered in *Chlamydia*. Southern blot analysis also demonstrated that insertion did not alter the loci surrounding *incA* and that the single intron insertion occurred at the predicted position within the chromosome. Finally, the ability to obtain *incA*::GII(*bla*) mutants from P_4_ enrichment cultures after plaquing for 14 days in the absence of selection indicates that the insertion is stable. One of these plaque isolates, DFCT4, was then passaged four additional times in the absence of selection prior to full genotypic and phenotypic analysis. No data suggesting the presence of *incA* revertants was seen with DFCT4. Whether this is due to the intrinsic stability of the insertion (at the DNA and RNA level), the absence of the *ltrA*, and/or the reduced activity of the Lt.LtrB intron at 37°C (optimal activity is ∼30°C [34]) remains unknown.

The modified TargeTron™ system reported in our study should be suitable for inactivation of other chlamydial genes, assuming they are non-essential. In *E. coli*, at least one intron insertion site is predicted to be present for every 130 bp of genomic sequence [35]. Our data from insertion predictions for 7500 bp of genomic DNA from *C. trachomatis* 434/Bu resulted in 81 high efficiency insertion sites (E-values less than 0.5), which would suggest that one insertion site should exist for every ∼100 bp of sequence, similar to that observed with *E. coli*. Based on the relative genome conservation between *Chlamydia* spp., we anticipate that similar insertion frequencies are present in other *C. trachomatis* serovars and *Chlamydia* spp. [36].

To allow for use of this platform outside of *C. trachomatis* LGV serovars and to allow for inactivation of multiple genes and gene complementation, we are pursuing alternative selection markers as well as markers suitable for mutant screens. Finally, as the GII intron normally carries a ∼1.8 kbp gene “cargo”, we also are testing the use of smaller selection cassettes to allow for modification of the intron to carry and insert gene cargos (cargo size with this system is limited due to the requirements of proper RNA folding for gene recognition and insertion). Ultimately, we anticipate that this newly adapted technology will open up avenues of chlamydial research not previously possible. Past research has been forced to study *Chlamydia* through the lens of the cell, using manipulation of the host cell environment to determine the importance of chlamydial factors during infection and greatly limiting the types of questions that could be asked. With this new platform and the growing list of plasmid-based gene delivery vehicles and forward-genetic approaches, the chlamydial field may now begin to assess gene function and contribution to disease through direct manipulation of the organism in line with the requirements of molecular Koch’s postulates [37]. In conclusion, our study further supports the genetic tractability of *Chlamydia* and further expands the list of genetic methods available for manipulating the chlamydial genome.

## Materials and Methods

### Ethics Statement

All recombinant DNA experiments were performed in accordance with NIH section III-D-1-a and were approved by the Southern Illinois University recombinant DNA committee.

### Culturing Conditions

All bacterial strains used in this study are listed in Table S1. Cell and *C. trachomatis* culture: Mouse fibroblast L2 cells (from [38]) were cultured in T75 culture flasks (BD Falcon) using Dulbecco’s Modified Eagle Medium (HyClone, Thermo Scientific™) with GlutaMAX™ (Gibco®, Life Technologies™) (DMEM) supplemented with 10% Fetal Bovine Serum (HyClone, Thermo Scientific™) and grown at 37°C with 5% CO_2_. Cells were routinely checked for mycoplasma contamination using PCR and degenerate primers to the 16S rRNA of *Mycoplasma* spp. (Stratagene). *C. trachomatis* were propagated in L2 cells and stored in Sucrose Phosphate Glycine buffer (SPG, 0.19 mM KH_2_PO_4_, 0.36 mM K_2_HPO_4,_ 0.245 mM L-glutamic acid, 10.9 mM sucrose) at −80°C. Stocks were titered using the Inclusion Forming Unit (IFU) assay.

#### Bacterial culture

*E. coli* strains were grown in Luria-Bertani (LB) broth or on LB agar plates supplemented with the appropriate antibiotics (kanamycin at 50 µg/ml, ampicillin at 100 µg/ml, and/or chloramphenicol at 20 µg/ml [all from Fisher BioReagents]) at 37°C.

### pDFTT3 Plasmid Construction

Plasmids were routinely passaged in *E. coli* DH5α. The base TargeTron™ vector pACD4K-C (Figure S1A) was obtained from Sigma. The kanamycin resistance RAM cassette was removed by digesting the vector with MluI (unless noted otherwise all molecular biology reagents are from Thermo Scientific™ Fermentas) followed by agarose gel purification (using the GeneJET Gel Extraction and Purification Kit, Thermo Scientific™) and ligation of the backbone vector using T4 DNA ligase. Ligated vector was transformed into *E. coli* and selected on LB agar chloramphenicol plates. Colonies were patched onto LB agar kanamycin plates and LB agar chloramphenicol plates and a chloramphenicol-resistant, kanamycin-sensitive clone was grown for plasmid purification (pDFTT1) using the GeneJET Plasmid Miniprep Kit (Thermo Scientific™). Loss of the RAM cassette was confirmed with primers GIIFtest and GIIRtest (all primer sequences are listed in Table S2). Primers CTL0655 proF and proR were then used to clone the 264 bp region upstream of the initiation codon of CTL0655 with 5′ XbaI and 3′ HindIII sites with Platinum Taq DNA Polymerase, High Fidelity (Life Technologies™) using genomic DNA from ACE051 (a plaque-purified clonal strain of *C. trachomatis* 434/Bu L2 from Anthony Maurelli, Uniformed Services University of the Health Sciences [39]). The *C. trachomatis* L2 434/Bu genome sequence (accession number NC_010287, [40]) was used for designing all PCR and Southern blot protocols. The PCR product was gel purified, digested with XbaI and HindIII, and ligated into similarly digested, gel purified pDFTT1 to clone the CTL0655 promoter upstream of the GII 5′ exon, creating pDFTT2. Ligation products were used for transformation and transformants were selected on LB agar chloramphenicol plates. Clones were screened for the insert using PCR and a positive clone was chosen for plasmid isolation and sequencing. pDFTT2 was then modified to confer ampicillin resistance to *Chlamydia* upon intron insertion by cloning the *bla* gene from pGFP::SW2 (kindly provided by Ian Clarke, University of Southampton, [9]) into the MluI site within the GII intron using primers blaF and blaR with Phusion High-Fidelity PCR Master Mix (Thermo Scientific™). PCR products were MluI digested, gel purified, and ligated into MluI digested and gel purified pDFTT2. Transformants carrying pDFTT2(*bla*) were selected on LB agar ampicillin/chloramphenicol plates and screened for insert orientation using primers blaF and GIIRtest. All vectors were sequence verified.

### Targeting the Intron to *incA*

The *incA* sequence from *C. trachomatis* 434/Bu was analyzed using the TargeTron™ algorithm (Sigma) for potential insertion sites. The predicted *incA* insertion site with the lowest E-value (0.191) and closest proximity to the 5′ ATG initiation codon was chosen. The primers 1608|1609s-IBS, 1608|1609s-EBS1d, 1608|1609s-EBS2, and EBS universal (Table S2) were used to retarget pDFTT3 as directed by the TargeTron™ manual. Briefly, a 350 bp product with 5′ HindIII and 3′ BsrGI sites was obtained, digested, and ligated into the similarly digested pDFTT2(*bla*) to create pDFTT3. Ligation products were transformed into *E. coli* DH5α. Clones were then isolated and sequence verified using primer T7 pro. The pDFTT3 vector map and sequence are reported in Figure S1B and S6, respectively.

### Creation of Site-specific, Insertionally-inactivated *incA*::GII(*bla*) Mutants

ACE051 was transformed with pDFTT3 using a calcium chloride protocol adapted from Wang *et. al* [9]. The steps were as follows (see Figure S7 for a transformation/selection method flow chart):

1. 1×10^7^ IFUs were thawed on ice and mixed by pipetting with 3 µg of pDFTT3 DNA and then diluted into molecular grade water and 5× CaCl_2_ buffer (50 mM Tris base, pH 7.4, 250 mM CaCl_2_) to yield a 1× CaCl_2_ solution in a 200 µl reaction volume. Samples were then left at room temperature for 30 minutes.
2. After 30 minutes, transformation samples were mixed with 12 ml of SPG and used to infect confluent L2 cells grown in 6-well tissue culture dishes (BD Falcon) at 2 ml/well. Infection was initiated by centrifugation at 545×g for 1 hour at room temperature in a clinical centrifuge.
3. After 1 hour, the SPG was removed from the cells and replaced with 2 ml DMEM/FBS and the cells were incubated at 37°C, 5% CO_2_ for 12 hours post infection (PI) (T_0_ = start of centrifugation).
4. At 12 hours PI, the medium was removed and replaced with 2 ml DMEM/FBS supplemented with 1 µg/ml ampicillin (Fisher BioReagents). The infection was allowed to proceed until 40–44 hours PI, at which point cells contained inclusions with aberrant bodies.
5. Sterile glass beads were added to the well and the plate was rocked to dislodge attached cells. Medium was collected in a 50 ml conical and the sample was sonicated at 20% duty for 20 seconds using a 1/8 inch probe to release EBs (Passage P_0_). Samples were then centrifuged at 10000×g for 20 minutes at 4°C. The pellet was retained and suspended in 2.5 ml SPG via vortexing and pipetting. The SPG stock was then used immediately to infect a confluent L2 cell monolayer in a T75 flask (P_1_). Cells were infected via rocking for two hours at 37°C, 5% CO_2_. The medium was then removed and 20 ml complete infection medium (DMEM/FBS, 0.2 µg/ml cyclohexamide [Sigma], 1× non-essential amino acids [HyClone, Thermo Scientific™]) supplemented with 1 µg/ml ampicillin was added to the flask, which was then incubated for 40–44 hours PI (T_0_ = start of rocking). Only inclusions containing aberrant bodies were readily found at this stage (apparent MOI <0.1).
6. Flasks were harvested using glass beads to remove the cells and the medium was collected in a 50 ml conical. Samples were sonicated to release EBs using a 1/8 inch probe set at 30% duty for 20 seconds. EBs were then pelleted by centrifuging the sample for 20 minutes at 10000×g at 4°C. Pellets were suspended and used to infect a T75 (as described in step 5 for P_1_) to generate P_2._ Healthy, active inclusions were visible during P_2_ with an apparent MOI of <0.01.
7. P_2_ was harvested (as described for P_1_) and used for P_3_ with the exception that the medium was supplemented with 5 µg/ml ampicillin instead of 1 µg/ml. P_3_ infected cells typically appeared to be infected at an MOI of ≤1. P_3_ EBs were harvested and used for an additional enrichment round in a T75 flask (using 1/4 of the SPG harvest from P_3_ for infection) with complete medium supplemented with 1 µg/ml ampicillin.
8. P_4_ was harvested, titered in L2 cells using the IFU assay, and then used to generate plaque-purified clones (performed as previously described [41]). Plaque-purified clones were obtained in the presence or absence of 1 µg/ml ampicillin. Plaque dishes were incubated for 14 days with additional medium overlays added on days 5 and 10 (with or without drug to match the original treatment).
9. Plaque-purified clones were serially expanded starting with a 6-well dish (subsequent drug treatment corresponded to plaque selection conditions) and moving through a T25 flask, a T75 flask, and finally into eight T175 flasks. T175 expanded EBs were harvested, titered, and stocked in SPG at −80°C for subsequent experiments.

### Genotyping Analyses of Mutants

Genomic DNA from clones and the wild-type parental strain was obtained from chlamydial stocks using the DNeasy Blood & Tissue Kit (Qiagen) as directed by the manufacturer.

#### PCR genotyping

PCR screening reactions were performed using the primers listed in Table S2 and 2× PCR Master Mix. Primers were used at 0.5 µM and template was added at 50 ng for genomic templates and 10 ng for plasmid templates. Reaction conditions for all PCR reactions performed in this study are listed in Table S3.

#### Sequencing of *incA*::GII(*bla*) insertion sites

The *incA* region was amplified from ACE051, DFTT3, or DFTT4 using primers incAseqF and incAseqR, which contain 5′ StuI restriction sites, using Phusion High-Fidelity PCR Master Mix. PCR products were gel purified and digested with StuI. StuI digested PCR products were then ligated into SmaI-digested pUC18. Ligation products were transformed into *E. coli* and selected on LB agar ampicillin plates. Transformants carrying the desired inserts were identified by blue-white screening and verified by PCR. The recombinant pUC18 plasmids (pUC18*incA*, pUC18*incA*::GII(*bla*)^DFTT3^, and pUC18*incA*::GII(*bla*)^DFTT4^) were isolated and sent for Sanger sequencing. Vectors were sequenced with primers pUCF, pUCR, GIIFtest, GIIRtest, and blaF2.

#### Southern blot analysis

2 µg of genomic DNA was digested with either PstI, SphI, or SacI and separated on 0.7% agarose gels. Gels were stained with ethidium bromide and visualized under UV transillumination. DNA was then transferred to positively charged nylon membranes and probed with DIG-labeled probes against *incA* or *bla*. DIG-probes were constructed using PCR to incorporate the DIG-11-dUTP label as directed by the manufacturer (Rosche). Blots were probed overnight at 42°C and high-stringency washes were performed at 65°C. Bound probes were detected using the alkaline phosphatase anti-DIG antibody-based DIG Nucleic Acid Detection Kit from Rosche. Antibody binding was localized using the precipitating chromogenic substrate nitroblue tetrazolium chloride. Reactions were halted by the addition of deionized, distilled water and blots were photographed using a BioRad ChemiDoc MP system.

### IncA and MOMP Western Blotting

24-well tissue culture plates were seeded with fibroblast L2 cells and incubated until the monolayers were ∼90% confluent. Three wells were then treated with trypsin to detach cells for trypan blue counting. Wells were then infected at an MOI of ten with ACE051, DFTT3, or DFTT4 diluted in 1 ml of complete infection medium (without ampicillin). Infection was initiated by centrifugation at 545×g for 1 hour at room temperature. Cells were then incubated at 37°C, 5% CO_2_ for 24 hours PI. Cells were visually inspected and photographed at 400× under phase contrast using a Leica DMIL inverted microscope fitted with a Leica EC3 camera using Leica LASV4.1 software for image analysis prior to processing for Western blotting.

Medium from wells used for Western blotting was aspirated and cells were washed with PBS. 250 µl of 1× Laemmli buffer (with 358 mM β-mercaptoethanol) was then added to each well and a pipet was used to scrape each well. Laemmli-suspended samples were transferred to an eppendorf tube, briefly sonicated, and heated at 95°C for 5 minutes. Samples were then run on 12% SDS-PAGE gels. Replicate gels were run to allow for Coomassie Brilliant Blue staining to ensure equal loading of samples, anti-MOMP Western blotting, and anti-IncA Western blotting. For Western blots, samples were transferred to nitrocellulose membranes and blocked with 5% milk Tris-buffered saline (MTBS). After blocking, blots were probed overnight at 4°C with either rabbit anti-IncA antibodies (kindly provided by Dr. Raphael Valdivia, Duke University [42]) or mouse anti-MOMP antibodies (from Abcam, provided by Wiley Jenkins, Southern Illinois University) diluted in MTBS at 1∶200 or 1∶1000, respectively. Blots were then washed with TBS/Tween-20 (0.05% V/V) and then probed with secondary goat anti-rabbit-IgG-HRP-conjugated antibody (Thermo Scientific™ Pierce, IncA blots) or anti-mouse-IgG-HRP-conjugated antibodies (Millipore, MOMP blots) diluted in MTBS at 1∶5000 for 1 hour at room temperature. Blots were then washed in TBS/Tween-20 followed by TBS and subsequently incubated with chemiluminescent substrate (SuperSignal WestPico, Thermo Scientific™ Pierce). Bands were visualized using a BioRad ChemiDoc MP.

### Light and Immunofluorescence (IF) Microscopy Analysis of *incA*::GII(*bla*) Mutants

Cells were seeded and infected in 24 well tissue culture dishes as described above for Western blotting except that wells contained acid-treated glass coverslips for IF microscopy. Replicate, non-coverslip wells were viewed under phase contrast at 400× to obtain light micrographs and to verify chlamydial infection. Cells were infected at an MOI of 0.01 (for 24 and 48 hour time points), 5 (for 24 and 48 hour time points), or 10 (for 24 hour time points). Cells were then fixed and permeablized for IF microscopy as previously described. *Chlamydia* were immunodetected using a primary mouse anti-MOMP antibody (Abcam) followed by a secondary donkey-anti-mouse IgG antibody conjugated to Texas Red (Thermo Scientific™). After antibody staining, cells were stained with DAPI and coverslips were mounted onto glass slides using ProLong Gold anti-Fade (Life Technologies™) prior to viewing with a Leica DM4000 fluorescent microscope fitted with a QImaging QiClick Mono (QImaging). Images were taken at 630× magnification under oil immersion and processed using QImaging software (QImaging).

## Supporting Information

Figure S1 **Vector maps of the commercial TargeTron™ vector pACD4K-C and the modified, chlamydial vector pDFTT3.** The commercial vector pACD4K-K (A) was modified for use in *Chlamydia* (pDFTT3, B) by removing the kan RAM cassette (in blue/purple) via MluI digestion and replacing the cassette with the *bla* marker from pGFP::SW2. The CTL0655 promoter region was then placed in front of the intron 5′exon between the XbaI and HindIII sites. Finally, the intron was retargeted for *incA* by exchanging the region between the BsrGI and HindIII sites with the appropriate targeting sequence. The δ sequence site is located adjacent to the EBS1 site and is notated as EBS1d on both plasmid maps (where d = δ).
(TIFF)

Figure S2 **Map of the** ***incA*** **::GII(** ***bla*** **) insertion site.** The *incA*::GII(*bla*) from DFCT3 and DFCT4 was PCR amplified and cloned into pUC18 for sequencing to map the intron insertion site. The intron disrupts the *incA* open reading frame between bps 108 and 109 (position 1 is the A in ATG). The full locus sequencing results are shown in Figure S5. The presence of premature stop codons are designated with red asterisks and the intron sequence is shown in red. The location of the incAF primer is shown in green.
(TIFF)

Figure S3 **PCR analysis of plaque-purified clones obtained with or without ampicillin selection.** Plaque dishes were infected with ∼50 IFU and grown for 14 days in the presence or absence of ampicillin. After 14 days, plaques were picked and expanded once in 6 well tissue culture dishes with or without ampicillin (matching the plaque conditions). EBs were harvested and genomic DNA was isolated for PCR analysis. PCR reaction products were separated on 1% agarose gels and detected using ethidium bromide staining and UV transillumination. Images have been reversed to increase contrast. Isolates plaqued in the absence of ampicillin are shown in A while isolates plaqued in the presence of ampicillin are shown in B. Reaction numbers correspond to the reactions shown in Figure 1.
(TIFF)

Figure S4 **Demonstration of ampicillin resistance of DFCT3 and DFCT4 versus ACE051 using the plaque assay.** L2 cells were grown until confluent in 60 mm cell culture dishes and then infected with 10^5^ IFU for 2 hours via rocking. Infection medium was then removed and plaque overlay containing no ampicillin or increasing amounts of ampicillin was added. Strains are listed at the top of each column and the concentration of ampicillin (µg/ml) is listed to the left of each row. Plaque dishes were incubated for 14 days, stained with neutral red, and then overlays were removed to reveal the presence or absence of intact cell monolayers.
(TIFF)

Figure S5 **Sanger sequencing results for the** ***incA*** **::GII (** ***bla*** **) locus.** The *incA* locus was amplified from ACE051, DFCT3, and DFCT4 and cloned into pUC18 for Sanger sequencing. The wild-type locus matched the sequence published for *C. trachomatis* 434/Bu (not shown) [40]. The sequencing results for the *incA*::GII(*bla*) locus for DCT3 and DFCT4 were identical. The *incA* ORF is shown in blue, the GII intron sequence is in red, and the *bla* ORF is in green.
(PDF)

Figure S6 **Vector sequence for pDFTT3.** The retargeted intron sequences are shown as N within the sequence file. The actual sequences at these positions are shown in the file header under IBS (5′ exon) inc, EBS2 inc, and EBS1d inc.
(PDF)

Figure S7 **Outline of the transformation and selection protocol used to obtain mutants.**
(TIFF)

Table S1 **Cell and bacterial strains.**
(XLSX)

Table S2 **Primers.**
(XLSX)

Table S3 **PCR reaction conditions.**
(XLSX)
