# Supplementary figures and images for: Site-Specific, Insertional Inactivation of incA in Chlamydia trachomatis Using a Group II Intron
Source: PLoS One. 2013 Dec 31;8(12):e83989. doi: 10.1371/journal.pone.0083989 (PMC3877132; doi:10.1371/journal.pone.0083989)

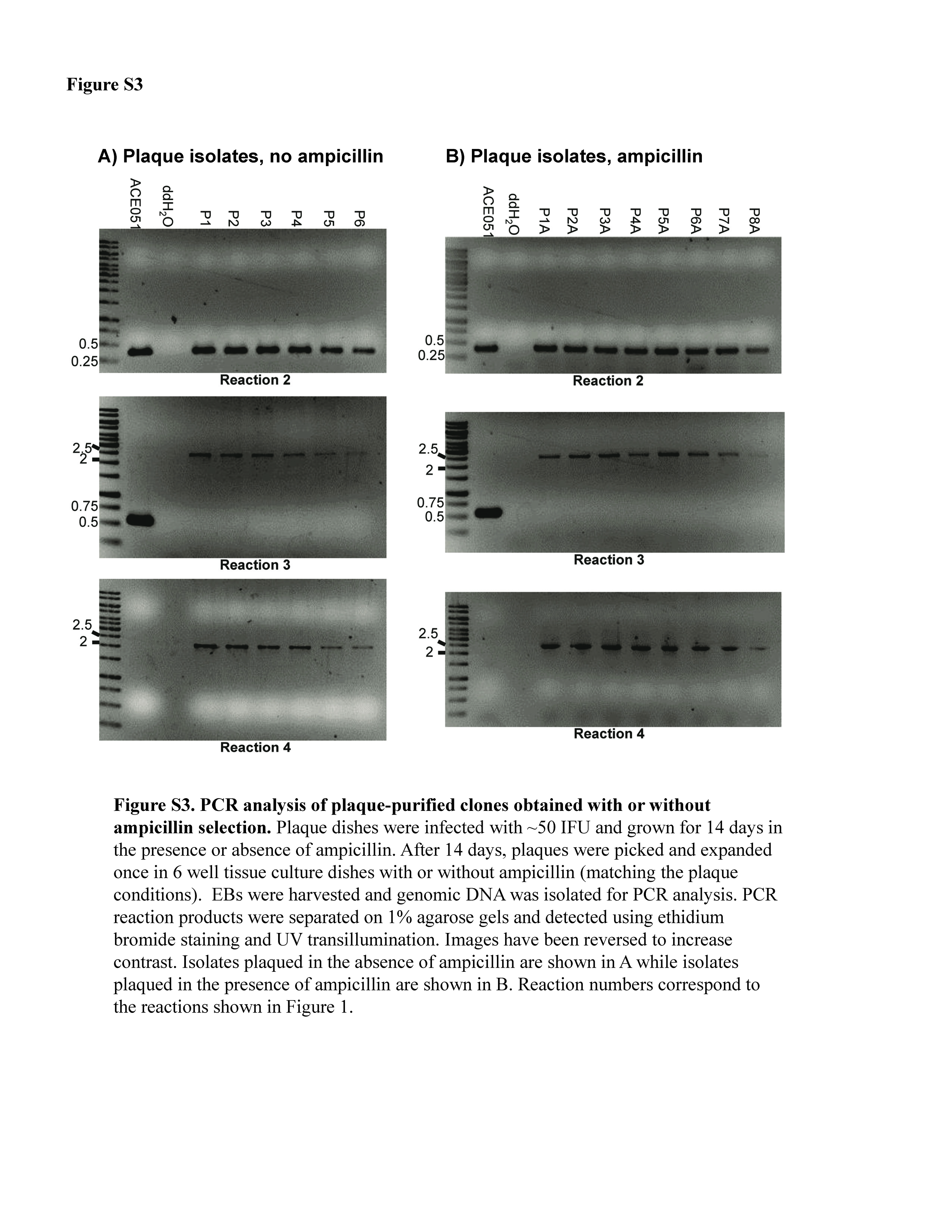

Supplement: Figure S3 — PCR analysis of plaque-purified clones obtained with or without ampicillin selection. Plaque dishes were infected with ∼50 IFU and grown for 14 days in the presence or absence of ampicillin. After 14 days, plaques were picked and expanded once in 6 well tissue culture dishes with or without ampicillin (matching the plaque conditions). EBs were harvested and genomic DNA was isolated for PCR analysis. PCR reaction products were separated on 1% agarose gels and detected using ethidium bromide staining and UV transillumination. Images have been reversed to increase contrast. Isolates plaqued in the absence of ampicillin are shown in A while isolates plaqued in the presence of ampicillin are shown in B. Reaction numbers correspond to the reactions shown in Figure 1. (TIFF) [file pone.0083989.s003.tiff]

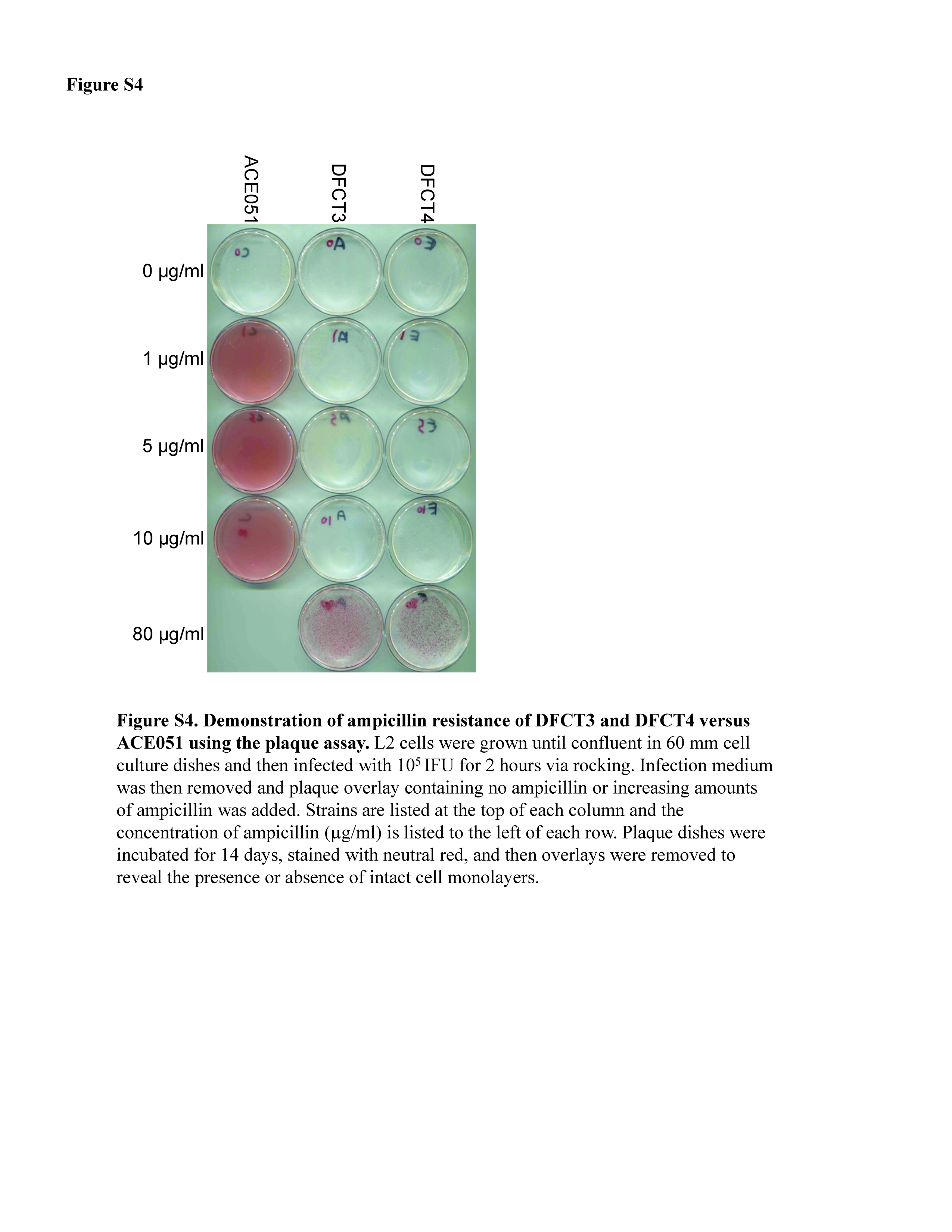

Supplement: Figure S4 — Demonstration of ampicillin resistance of DFCT3 and DFCT4 versus ACE051 using the plaque assay. L2 cells were grown until confluent in 60 mm cell culture dishes and then infected with 105 IFU for 2 hours via rocking. Infection medium was then removed and plaque overlay containing no ampicillin or increasing amounts of ampicillin was added. Strains are listed at the top of each column and the concentration of ampicillin (µg/ml) is listed to the left of each row. Plaque dishes were incubated for 14 days, stained with neutral red, and then overlays were removed to reveal the presence or absence of intact cell monolayers. (TIFF) [file pone.0083989.s004.tiff]
