# Supplementary material for: Site-Specific, Insertional Inactivation of incA in Chlamydia trachomatis Using a Group II Intron
Source: PLoS One. 2013 Dec 31;8(12):e83989. doi: 10.1371/journal.pone.0083989 (PMC3877132; doi:10.1371/journal.pone.0083989)
Supplement: Figure S5 — Sanger sequencing results for the incA ::GII ( bla ) locus. The incA locus was amplified from ACE051, DFCT3, and DFCT4 and cloned into pUC18 for Sanger sequencing. The wild-type locus matched the sequence published for C. trachomatis 434/Bu (not shown) [40]. The sequencing results for the incA::GII(bla) locus for DCT3 and DFCT4 were identical. The incA ORF is shown in blue, the GII intron sequence is in red, and the bla ORF is in green. (PDF) [file pone.0083989.s005.pdf]

Figure S5

```
         10        20        30        40 incA   50        60        70        80        90       100
....|....|....|....|....|....|....|....|....|....|....|....|....|....|....|....|....|....|....|....|
GGATCTGGTTTCAGAGAACTTTAAATGAGAAAGTGCCTATGACAACGCCTACTCTAATCGTGACTCCTCCATCTCCCCCTGCACCTTCCTACTCAGCCAA

        110       120       130       140       150   GII 160       170       180       190       200
....|....|....|....|....|....|....|....|....|....|....|....|....|....|....|....|....|....|....|....|
TCGCGTACCTCAACCTTCTTTGATGGACAAAATTAAGAAAATAGCAGTGCGCCCAGATAGGGTGTTAAGTCAAGTAGTTTAAGGTACTACTCTGTAAGAT

        210       220       230       240       250       260       270       280       290       300
....|....|....|....|....|....|....|....|....|....|....|....|....|....|....|....|....|....|....|....|
AACACAGAAAACAGCCAACCTAACCGAAAAGCGAAAGCTGATACGGGAACAGAGCACGGTTGGAAAGCGATGAGTTACCTAAAGACAATCGGGTACGACT

        310       320       330       340       350       360       370       380       390       400
....|....|....|....|....|....|....|....|....|....|....|....|....|....|....|....|....|....|....|....|
GAGTCGCAATGTTAATCAGATATAAGGTATAAGTTGTGTTTACTGAACGCAAGTTTCTAATTTCGGTTTTCTTCCGATAGAGGAAAGTGTCTGAAACCTC

        410       420       430       440       450       460       470       480       490       500
....|....|....|....|....|....|....|....|....|....|....|....|....|....|....|....|....|....|....|....|
TAGTACAAAGAAAGGTAAGTTAGCTGCTATGACTTATCTGTTATCACCACATTTGTACAATCTGTAGGAGAACCTATGGGAACGAAACGAAAGCGATGCC

        510       520       530       540       550       560       570       580       590       600
....|....|....|....|....|....|....|....|....|....|....|....|....|....|....|....|....|....|....|....|
GAGAATCTGAATTTACCAAGACTTAACACTAACTGGGGATACCCTAAACAAGAATGCCTAATAGAAAGGAGGAAAAAGGCTATAGCACTAGAGCTTGAAA

        610       620       630       640       650       660       670       680       690       700
....|....|....|....|....|....|....|....|....|....|....|....|....|....|....|....|....|....|....|....|
ATCTTGCAAGGGTACGGAGTACTCGTAGTAGTCTGAGAAGGGTAACGCCCTTTACATGGCAAAGGGGTACAGTTATTGTGTACTAAAATTAAAAATTGAT

        710       720       730       740       750       760       770       780       790       800
....|....|....|....|....|....|....|....|....|....|....|....|....|....|....|....|....|....|....|....|
TAGGGAGGAAAACCTCAAAATGAAACCAACAATGGCAATTTTAGAAAGAATCAGTAAAAATTCACAAGAAAATATAGACGAAGTTTTTACAAGACTTTAT

        810       820       830       840       850       860       870       880       890       900
....|....|....|....|....|....|....|....|....|....|....|....|....|....|....|....|....|....|....|....|
CGTTATCTTTTACGTCCAGATATTTATTACGTGGCGACGCGTAGGTTAATGTCATGATAATAATGGTTTCTTAGACGTCAGGTGGCACTTTTCGGGGAAA

        910       920       930       940       950       960       970       980       990      1000
....|....|....|....|....|....|....|....|....|....|....|....|....|....|....|....|....|....|....|....|
TGTGCGCGGAACCCCTATTTGTTTATTTTTCTAAATACATTCAAATATGTATCCGCTCATGAGACAATAACCCTGATAAATGCTTCAATAATATTGAAAA

       1010 bla  1020      1030      1040      1050      1060      1070      1080      1090      1100
....|....|....|....|....|....|....|....|....|....|....|....|....|....|....|....|....|....|....|....|
AGGAAGAGTATGAGTATTCAACATTTCCGTGTCGCCCTTATTCCCTTTTTTGCGGCATTTTGCCTTCCTGTTTTTGCTCACCCAGAAACGCTGGTGAAAG

       1110      1120      1130      1140      1150      1160      1170      1180      1190      1200
....|....|....|....|....|....|....|....|....|....|....|....|....|....|....|....|....|....|....|....|
TAAAAGATGCTGAAGATCAGTTGGGTGCACGAGTGGGTTACATCGAACTGGATCTCAACAGCGGTAAGATCCTTGAGAGTTTTCGCCCCGAAGAACGTTT

       1210      1220      1230      1240      1250      1260      1270      1280      1290      1300
....|....|....|....|....|....|....|....|....|....|....|....|....|....|....|....|....|....|....|....|
TCCAATGATGAGCACTTTTAAAGTTCTGCTATGTGGCGCGGTATTATCCCGTATTGACGCCGGGCAAGAGCAACTCGGTCGCCGCATACACTATTCTCAG

       1310      1320      1330      1340      1350      1360      1370      1380      1390      1400
....|....|....|....|....|....|....|....|....|....|....|....|....|....|....|....|....|....|....|....|
AATGACTTGGTTGAGTACTCACCAGTCACAGAAAAGCATCTTACGGATGGCATGACAGTAAGAGAATTATGCAGTGCTGCCATAACCATGAGTGATAACA

       1410      1420      1430      1440      1450      1460      1470      1480      1490      1500
....|....|....|....|....|....|....|....|....|....|....|....|....|....|....|....|....|....|....|....|
CTGCGGCCAACTTACTTCTGACAACGATCGGAGGACCGAAGGAGCTAACCGCTTTTTTGCACAACATGGGGGATCATGTAACTCGCCTTGATCGTTGGGA

       1510      1520      1530      1540      1550      1560      1570      1580      1590      1600
....|....|....|....|....|....|....|....|....|....|....|....|....|....|....|....|....|....|....|....|
ACCGGAGCTGAATGAAGCCATACCAAACGACGAGCGTGACACCACGATGCCTGTAGCAATGGCAACAACGTTGCGCAAACTATTAACTGGCGAACTACTT

       1610      1620      1630      1640      1650      1660      1670      1680      1690      1700
....|....|....|....|....|....|....|....|....|....|....|....|....|....|....|....|....|....|....|....|
ACTCTAGCTTCCCGGCAACAATTAATAGACTGGATGGAGGCGGATAAAGTTGCAGGACCACTTCTGCGCTCGGCCCTTCCGGCTGGCTGGTTTATTGCTG

       1710      1720      1730      1740      1750      1760      1770      1780      1790      1800
....|....|....|....|....|....|....|....|....|....|....|....|....|....|....|....|....|....|....|....|
ATAAATCTGGAGCCGGTGAGCGTGGGTCTCGCGGTATCATTGCAGCACTGGGGCCAGATGGTAAGCCCTCCCGTATCGTAGTTATCTACACGACGGGGAG
```

```
      1810      1820      1830      1840      1850      1860      1870      1880      1890      1900
....|....|....|....|....|....|....|....|....|....|....|....|....|....|....|....|....|....|....|....|
TCAGGCAACTATGGATGAACGAAATAGACAGATCGCTGAGATAGGTGCCTCACTGATTAAGCATTGGTAACTGTCAGACCAAGTTTACTCATATATACTT

      1910      1920      1930      1940      1950      1960      1970      1980      1990      2000
....|....|....|....|....|....|....|....|....|....|....|....|....|....|....|....|....|....|....|....|
TAGATTGATTTAAAACTTCATTTTTAATTTAAAAGGATCTAGGTGAAGATCCTTTTTGATAATCTCATGACCAAAATCCCTTAACGTGAGTTTTCGTTCC

      2010      2020      2030      2040      2050      2060      2070      2080      2090      2100
....|....|....|....|....|....|....|....|....|....|....|....|....|....|....|....|....|....|....|....|
ACACGCGTTGGGAAATGGCAATGATAGCGAAACAACGTAAAACTCTTGTTGTATGCTTTCATTGTCATCGTCACGTGATTCATAAACACAAGTGAATGTC

      2110      2120      2130      2140      2150      2160      2170      2180      2190      2200
....|....|....|....|....|....|....|....|....|....|....|....|....|....|....|....|....|....|....|....|
GACAGTGAATTTTTACGAACGAACAATAACAGAGCCGTATACTCCGAGAGGGGTACGTACGGTTCCCGAAGAGGGTGGTGCAAACCAGTCACAGTAATGT

      2210      2220      2230      2240      2250      2260      2270      2280      2290      2300
....|....|....|....|....|....|....|....|....|....|....|....|....|....|....|....|....|....|....|....|
GAACAAGGCGGTACCTCCCTACTTCACGCCATTGCCTCCCTAATTCTTATAGGCACAATAGGCTTTTTAGCTCTTTTGGGACATCTTGTTGGCTTTCTGA

      2310      2320      2330      2340      2350      2360      2370      2380      2390      2400
....|....|....|....|....|....|....|....|....|....|....|....|....|....|....|....|....|....|....|....|
TCGCTCCACAAATCACTATTGTTCTTCTTGCCCTATTCATTATCTCATTAGCAGGGAATGCTCTTTATCTACAGAAAACCGCTAATCTACATCTATACCA

      2410      2420      2430      2440      2450      2460      2470      2480      2490      2500
....|....|....|....|....|....|....|....|....|....|....|....|....|....|....|....|....|....|....|....|
GGATCTGCAAAGAGAAGTTGGGTCTCTAAAAGAAATTAATTTCATGCTGAGCGTTCTACAGAAAGAATTTCTTCATTTATCTAAAGAATTTGCAACGACA

      2510      2520      2530      2540      2550      2560      2570      2580      2590      2600
....|....|....|....|....|....|....|....|....|....|....|....|....|....|....|....|....|....|....|....|
TCTAAAGACCTCTCTGCTGTATCTCAAGATTTTTATTCTTGTTTGCAAGGATTTAGAGATAACTATAAAGGTTTTGAATCTCTTTTGGATGAGTATAAAA

      2610      2620      2630      2640      2650      2660      2670      2680      2690      2700
....|....|....|....|....|....|....|....|....|....|....|....|....|....|....|....|....|....|....|....|
ACTCTACAGAAGAAATGCGCAAACTTTTTTCGCAAGAAATCATAGCAGATCTTAAAGGCTCTGTTGCCTCATTAAGAGAGGAAATCCGATTCCTAACCCC

      2710      2720      2730      2740      2750      2760      2770      2780      2790      2800
....|....|....|....|....|....|....|....|....|....|....|....|....|....|....|....|....|....|....|....|
ATTAGCAGAAGAAGTTCGCCGATTAGCGCATAACCAGCAATCATTAACAGTGGTTATTGAAGAATTAAAAACAATTCGTGATAGCTTACGAGATGAAATT

      2810      2820      2830      2840      2850      2860      2870      2880      2890      2900
....|....|....|....|....|....|....|....|....|....|....|....|....|....|....|....|....|....|....|....|
GGACAACTTTCACAACTTTCTAAAACTCTTACCAGTCAAATTGCATTACAACGAAAAGAGAGCTCAGATCTGTGTTCCCAGATAAGAGAGACGCTCTCCT

      2910      2920      2930      2940      2950      2960      2970      2980      2990      3000
....|....|....|....|....|....|....|....|....|....|....|....|....|....|....|....|....|....|....|....|
CCCCCAGAAAGTCTGCATCACCCTCTACAAAAAGCTCCTAGTCTTAGGAGCTTTTTGCAATGCAAAACATAACACCCTGTAAAAGGCTGCAGAACTAGTC

      3010      3020      3030      3040
....|....|....|....|....|....|....|....|...
TACTCATCCTAACTGCACACAGGGTGCCAATAGTAACGGAACT
```

Figure S5. Sanger sequencing results for the *incA*::GII(*bla*) locus. The *incA* locus was amplified from ACE051, DFCT3, and DFCT4 and cloned into pUC18 for Sanger sequencing. The wild-type locus matched the sequence published for *C. trachomatis* 434/Bu (not shown) [40]. The sequencing results for the *incA*::GII(*bla*) locus for DCT3 and DFCT4 were identical. The *incA* ORF is shown in blue, the GII intron sequence is in red, and the *bla* ORF is in green.
