# Supplementary material for: Site-Specific, Insertional Inactivation of incA in Chlamydia trachomatis Using a Group II Intron
Source: PLoS One. 2013 Dec 31;8(12):e83989. doi: 10.1371/journal.pone.0083989 (PMC3877132; doi:10.1371/journal.pone.0083989)
Supplement: Figure S6 — Vector sequence for pDFTT3. The retargeted intron sequences are shown as N within the sequence file. The actual sequences at these positions are shown in the file header under IBS (5′ exon) inc, EBS2 inc, and EBS1d inc. (PDF) [file pone.0083989.s006.pdf]

Figure S6

```
LOCUS       pDFTT3           7727 bp
FEATURES             Location/Qualifiers
     CDS             585..1497
                     /gene="p15A ori"
     CDS             1741..1762
                     /gene="T7 pro"
     CDS             1793..2055
                     /gene="CTL0655"
     misc_feature    2075..2086
                     /gene="IBS (5' exon) inc"
                     /product="aaagaaCatagca"
     CDS             2087..4167
                     /gene="intron  RNA"
     misc_signal     2087..4167
                     /label=insertion
                     /product="region inserted into target"
     misc_feature    2306..2314
                     /gene="EBS2 inc"
                     /product="gTTttcttc"
     misc_feature    2363..2370
                     /gene="EBS1d inc"
                     /product="gctgctat"
     CDS             2950..3810
                     /gene="bla"
     CDS             4168..4177
                     /gene="3' exon"
     CDS             4414..6213
                     /gene="ltrA"
     CDS             6349..6639
                     /gene="T1/T2 tran term"
     CDS             complement(join(7290..7727,1..219))
                     /gene="cat"
ORIGIN
        1 gaattccgga tgagcattca tcaggcgggc aagaatgtga ataaaggccg gataaaactt
       61 gtgcttattt ttctttacgg tctttaaaaa ggccgtaata tccagctgaa cggtctggtt
      121 ataggtacat tgagcaactg actgaaatgc ctcaaaatgt tctttacgat gccattggga
      181 tatatcaacg gtggtatatc cagtgatttt tttctccatt ttagcttcct tagctcctga
      241 aaatctcgat aactcaaaaa atacgcccgg tagtgatctt atttcattat ggtgaaagtt
      301 ggaacctctt acgtgccgat caacgtctca ttttcgccaa aagttggccc agggcttccc
      361 ggtatcaaca gggacaccag gatttattta ttctgcgaag tgatcttccg tcacaggtat
      421 ttattcggcg caaagtgcgt cgggtgatgc tgccaactta ctgatttagt gtatgatggt
      481 gtttttgagg tgctccagtg gcttctgttt ctatcagctg tccctcctgt tcagctactg
      541 acggggtggt gcgtaacggc aaaagcaccg ccggacatca gcgctagcgg agtgtatact
      601 ggcttactat gttggcactg atgagggtgt cagtgaagtg cttcatgtgg caggagaaaa
      661 aaggctgcac cggtgcgtca gcagaatatg tgatacagga tatattccgc ttcctcgctc
      721 actgactcgc tacgctcggt cgttcgactg cggcgagcgg aaatggctta cgaacggggc
      781 ggagatttcc tggaagatgc caggaagata cttaacaggg aagtgagagg gccgcggcaa
```

841 agccgttttt ccataggctc cgcccccctg acaagcatca cgaaatctga cgctcaaatc
901 agtggtggcg aaacccgaca ggactataaa gataccaggc gtttccccct ggcggctccc
961 tcgtgcgctc tcctgttcct gcctttcggt ttaccggtgt cattccgctg ttatggccgc
1021 gtttgtctca ttccacgcct gacactcagt tccgggtagg cagttcgctc caagctggac
1081 tgtatgcacg aaccccccgt tcagtccgac cgctgcgcct tatccggtaa ctatcgtctt
1141 gagtccaacc cggaaagaca tgcaaaagca ccactggcag cagccactgg taattgattt
1201 agaggagtta gtcttgaagt catgcgccgg ttaaggctaa actgaaagga caagttttgg
1261 tgactgcgct cctccaagcc agttacctcg gttcaaagag ttggtagctc agagaacctt
1321 cgaaaaaccg ccctgcaagg cggttttttc gttttcagag caagagatta cgcgcagacc
1381 aaaacgatct caagaagatc atcttattaa tcagataaaa tatttctagc tagatttcag
1441 tgcaatttat ctcttcaaat gtagcacctg aagtcagccc catacgatat aagttgtaat
1501 tctcatgttt gacagcttat catcgataag ctcaaggaga tggcgcccaa cagtcccccg
1561 gccacggggc ctgccaccat acccacgccg aaacaagcgc tcatgagccc gaagtggcga
1621 gcccgatctt ccccatcggt gatgtcggcg atataggcgc cagcaaccgc acctgtggcg
1681 ccggtgatgc cggccacgat gcgtccggcg tagaggatcg agatctcgat cccgcgaaat
1741 taatacgact cactataggg gaattgtgag cggataacaa ttcccctcta gaggtaccgg
1801 atcctccaaa ttattcctta catgaatttt ttgtctgagc gactttctcc cattgaaaaa
1861 gattttctta aacaaaacgt gctttacttc ttgcagaaaa atcggtaaac ttgccgtttc
1921 gtctaggcag actcgtccgc gtcttttttc aaaactccct tttaggaag tttttgaagg
1981 cgttcctcag attttcccga gttggaggag actggccggc actacaagct catatcaagg
2041 taaggaaaga tttccaagct tataattatc cttannnnnc nnnnnngtgc gcccagatag
2101 ggtgttaagt caagtagttt aaggtactac tctgtaagat aacacagaaa acagccaacc
2161 taaccgaaaa gcgaaagctg atacgggaac agagcacggt tggaaagcga tgagttacct
2221 aaagacaatc gggtacgact gagtcgcaat gttaatcaga tataaggtat aagttgtgtt
2281 tactgaacgc aagtttctaa tttcgnttnn nnnncgatag aggaaagtgt ctgaaacctc
2341 tagtacaaag aaaggtaagt tannnnnnnn gacttatctg ttatcaccac atttgtacaa
2401 tctgtaggag aacctatggg aacgaaacga aagcgatgcc gagaatctga atttaccaag
2461 acttaacact aactggggat accctaaaca agaatgccta atagaaagga ggaaaaaggc
2521 tatagcacta gagcttgaaa atcttgcaag ggtacggagt actcgtagta gtctgagaag
2581 ggtaacgccc tttacatggc aaaggggtac agttattgtg tactaaaatt aaaaattgat
2641 tagggaggaa aacctcaaaa tgaaaccaac aatggcaatt ttagaaagaa tcagtaaaaa
2701 ttcacaagaa aatatagacg aagtttttac aagactttat cgttatcttt tacgtccaga
2761 tatttattac gtggcgacgc gtaggttaat gtcatgataa taatggtttc ttagacgtca
2821 ggtggcactt ttcggggaaa tgtgcgcgga acccctattt gtttattttt ctaaatacat
2881 tcaaatatgt atccgctcat gagacaataa ccctgataaa tgcttcaata atattgaaaa
2941 aggaagagta tgagtattca acatttccgt gtcgccctta ttcccttttt tgcggcattt
3001 tgccttcctg tttttgctca cccagaaacg ctggtgaaag taaaagatgc tgaagatcag
3061 ttgggtgcac gagtgggtta catcgaactg gatctcaaca gcggtaagat ccttgagagt
3121 tttcgccccg aagaacgttt tccaatgatg agcactttta aagttctgct atgtggcgcg
3181 gtattatccc gtattgacgc cgggcaagag caactcggtc gccgcataca ctattctcag
3241 aatgacttgg ttgagtactc accagtcaca gaaaagcatc ttacggatgg catgacagta
3301 agagaattat gcagtgctgc cataaccatg agtgataaca ctgcggccaa cttacttctg
3361 acaacgatcg gaggaccgaa ggagctaacc gcttttttgc acaacatggg ggatcatgta
3421 actcgccttg atcgttggga accggagctg aatgaagcca taccaaacga cgagcgtgac
3481 accacgatgc ctgtagcaat ggcaacaacg ttgcgcaaac tattaactgg cgaactactt
3541 actctagctt cccggcaaca attaatagac tggatggagg cggataaagt tgcaggacca
3601 cttctgcgct cggcccttcc ggctggctgg tttattgctg ataaatctgg agccggtgag
3661 cgtgggtctc gcggtatcat tgcagcactg gggccagatg gtaagccctc ccgtatcgta
3721 gttatctaca cgacggggag tcaggcaact atggatgaac gaaatagaca gatcgctgag

3781 ataggtgcct cactgattaa gcattggtaa ctgtcagacc aagtttactc atatatactt
3841 tagattgatt taaaacttca tttttaattt aaaaggatct aggtgaagat cctttttgat
3901 aatctcatga ccaaaatccc ttaacgtgag ttttcgttcc acacgcgttg ggaaatggca
3961 atgatagcga aacaacgtaa aactcttgtt gtatgctttc attgtcatcg tcacgtgatt
4021 cataaacaca agtgaatgtc gacagtgaat ttttacgaac gaacaataac agagccgtat
4081 actccgagag gggtacgtac ggttcccgaa gagggtggtg caaaccagtc acagtaatgt
4141 gaacaaggcg gtacctccct acttcaccat atcattttct gcagccccct agaaataatt
4201 ttgtttaact ttaagaagga gatatacata tatggctaga tcgtccattc cgacagcatc
4261 gccagtcact atggcgtgct gctagcgcta tatgcgttga tgcaatttct atgcactcgt
4321 agtagtctga gaagggtaac gccctttaca tggcaaaggg gtacagttat tgtgtactaa
4381 aattaaaaat tgattaggga ggaaaacctc aaaatgaaac caacaatggc aattttagaa
4441 agaatcagta aaaattcaca agaaaatata gacgaagttt ttacaagact ttatcgttat
4501 cttttacgtc cagatattta ttacgtggcg tatcaaaatt tatattccaa taaaggagct
4561 tccacaaaag gaatattaga tgatacagcg gatggcttta gtgaagaaaa aataaaaaag
4621 attattcaat ctttaaaaga cggaacttac tatcctcaac ctgtacgaag aatgtatatt
4681 gcaaaaaaga attctaaaaa gatgagacct ttaggaattc caactttcac agataaattg
4741 atccaagaag ctgtgagaat aattcttgaa tctatctatg aaccggtatt cgaagatgtg
4801 tctcacggtt ttagacctca acgaagctgt cacacagctt tgaaaacaat caaaagagag
4861 tttggcggcg caagatggtt tgtggaggga gatataaaag gctgcttcga taatatagac
4921 cacgttacac tcattggact catcaatctt aaaatcaaag atatgaaaat gagccaattg
4981 atttataaat ttctaaaagc aggttatctg gaaaactggc agtatcacaa aacttacagc
5041 ggaacacctc aaggtggaat tctatctcct cttttggcca acatctatct tcatgaattg
5101 gataagtttg ttttacaact caaaatgaag tttgaccgag aaagtccaga aagaataaca
5161 cctgaatatc gggagctcca caatgagata aaaagaattt ctcaccgtct caagaagttg
5221 gagggtgaag aaaaagctaa agttctttta gaatatcaag aaaaacgtaa aagattaccc
5281 acactcccct gtacctcaca gacaaataaa gtattgaaat acgtccggta tgcggacgac
5341 ttcattatct ctgttaaagg aagcaaagag gactgtcaat ggataaaaga acaattaaaa
5401 ctttttattc ataacaagct aaaaatggaa ttgagtgaag aaaaaacact catcacacat
5461 agcagtcaac ccgctcgttt tctgggatat gatatacgag taaggagatc tggaacgata
5521 aaacgatctg gtaaagtcaa aaagagaaca ctcaatggga gtgtagaact ccttattcct
5581 cttcaagaca aaattcgtca atttattttt gacaagaaaa tagctatcca aaagaaagat
5641 agctcatggt ttccagttca caggaaatat cttattcgtt caacagactt agaaatcatc
5701 acaatttata attctgaact ccgcgggatt tgtaattact acggtctagc aagtaatttt
5761 aaccagctca attattttgc ttatcttatg gaatacagct gtctaaaaac gatagcctcc
5821 aaacataagg gaacactttc aaaaaccatt tccatgttta aagatggaag tggttcgtgg
5881 gggatcccgt atgagataaa gcaaggtaag cagcgccgtt attttgcaaa ttttagtgaa
5941 tgtaaatccc cttatcaatt tacggatgag ataagtcaag ctcctgtatt gtatggctat
6001 gcccggaata ctcttgaaaa caggttaaaa gctaaatgtt gtgaattatg tgggacgtct
6061 gatgaaaata cttcctatga aattcaccat gtcaataagg tcaaaaatct taaaggcaaa
6121 gaaaaatggg aaatggcaat gatagcgaaa caacgtaaaa ctcttgttgt atgctttcat
6181 tgtcatcgtc acgtgattca taaacacaag tgaatgtcga gcacccgttc tcggagcact
6241 gtccgaccgc tttggccgcc gcccagtcct gctcgcttcg ctacttggag ccactatcga
6301 ctacgcgatc atggcgacca cacccgtcct gtggatcgcc aagctcgccg atggtagtgt
6361 ggggtctccc catgcgagag tagggaactg ccaggcatca aataaaacga aaggctcagt
6421 cgaaagactg ggcctttcgt tttatctgtt gtttgtcggt gaacgctctc ctgagtagga
6481 caaatccgcc gggagcggat ttgaacgttg cgaagcaacg gcccggaggg tggcgggcag
6541 gacgcccgcc ataaactgcc aggcatcaaa ttaagcagaa ggccatcctg acggatggcc
6601 tttttgcgtt tctacaaact cttcctgtcg tcatatctac aagccatccc cccacagata
6661 cggtaaacta gcctcgtttt tgcatcagga aagcagaacg ccatgagcgg cctcatttct

```
     6721 tattctgagt tacaacagtc cgcaccgctg tccggtagct ccttccggtg ggcgcggggc
     6781 atgactatcg tcgccgcact tatgactgtc ttctttatca tgcaactcgt aggacaggtg
     6841 ccggcagcgc ccaacagtcc cccggccacg gggcctgcca ccatacccac gccgaaacaa
     6901 gcgccctgca ccattatgtt ccggatctgc atcgcaggat gctgctggct accctgtgga
     6961 acacctacat ctgtattaac gaagcgctaa ccgtttttat caggctctgg gaggcagaat
     7021 aaatgatcat atcgtcaatt attacctcca cggggagagc ctgagcaaac tggcctcagg
     7081 catttgagaa gcacacggtc acactgcttc cggtagtcaa taaaccggta aaccagcaat
     7141 agacataagc ggctatttaa cgaccctgcc ctgaaccgac gaccgggtcg aatttgcttt
     7201 cgaatttctg ccattcatcc gcttattatc acttattcag gcgtagcacc aggcgtttaa
     7261 gggcaccaat aactgcctta aaaaaattac gccccgccct gccactcatc gcagtactgt
     7321 tgtaattcat taagcattct gccgacatgg aagccatcac agacggcatg atgaacctga
     7381 atcgccagcg gcatcagcac cttgtcgcct tgcgtataat atttgcccat ggtgaaaacg
     7441 ggggcgaaga agttgtccat attggccacg tttaaatcaa aactggtgaa actcacccag
     7501 ggattggctg agacgaaaaa catattctca ataaaccctt tagggaaata ggccaggttt
     7561 tcaccgtaac acgccacatc ttgcgaatat atgtgtagaa actgccggaa atcgtcgtgg
     7621 tattcactcc agagcgatga aaacgtttca gtttgctcat ggaaaacggt gtaacaaggg
     7681 tgaacactat cccatatcac cagctcaccg tctttcattg ccatacg
//
```

Figure S6. Vector sequence for pDFTT3. The retargeted intron sequences are shown as N within the sequence file. The actual sequences at these positions are shown in the file header under IBS (5’ exon) inc, EBS2 inc, and EBS1d inc.
